# The effect of empagliflozin on growth differentiation factor 15 in patients with heart failure: a randomized controlled trial (Empire HF Biomarker)

**DOI:** 10.1186/s12933-022-01463-2

**Published:** 2022-02-27

**Authors:** Massar Omar, Jesper Jensen, Caroline Kistorp, Kurt Højlund, Lars Videbæk, Christian Tuxen, Julie H. Larsen, Camilla F. Andersen, Finn Gustafsson, Lars Køber, Morten Schou, Jacob Eifer Møller

**Affiliations:** 1 Department of Cardiology, Research Unit of Cardiology, Odense University Hospital, Odense, Denmark; 2 Steno Diabetes Center Odense, Odense University Hospital, Odense, Denmark; 3 Department of Cardiology, Herlev, Gentofte University Hospital, Herlev, Denmark; 4 Department of Endocrinology, Rigshospitalet, Copenhagen, Denmark; 5 Department of Cardiology, Bispebjerg, Frederiksberg University Hospital, Copenhagen, Denmark; 6 Department of Cardiology, Rigshospitalet, Copenhagen, Denmark; 7 Department of Clinical Medicine, University of Copenhagen, Copenhagen, Denmark; 8 Faculty of Health Sciences, University of Southern Denmark, Odense, Denmark

**Keywords:** HFrEF, SGLT2 inhibitors, GDF15, hsTNT, hsCRP

## Abstract

**Background:**

Plasma growth differentiation factor-15 (GDF-15) biomarker levels increase in response to inflammation and tissue injury, and increased levels of GDF-15 are associated with increased risk of mortality in patients with heart failure with reduced ejection fraction (HFrEF). Sodium-glucose cotransporter-2 (SGLT2) inhibitors, which improve outcome in HFrEF, have been shown to increase plasma GDF-15 in diabetic patients. We aimed to investigate the effect of empagliflozin on GDF-15 in HFrEF patients.

**Methods:**

This Empire HF Biomarker substudy was from the multicentre, randomized, double-blind, placebo-controlled Empire HF trial that included 190 patients from June 29, 2017, to September 10, 2019. Stable ambulatory HFrEF patients with ejection fraction of ≤ 40% were randomly assigned (1:1) to empagliflozin 10 mg once daily, or matching placebo for 12 weeks. Changes from baseline to 12 weeks in plasma levels of GDF-15, high-sensitive C-reactive protein (hsCRP), and high-sensitive troponin T (hsTNT) were assessed.

**Results:**

A total of 187 patients who were included in this study, mean age was 64 ± 11 years; 85% male, 12% with type 2 diabetes, mean ejection fraction 29 ± 8, with no differences between the groups. Baseline median plasma GDF-15 was 1189 (918–1720) pg/mL with empagliflozin, and 1299 (952–1823) pg/mL for placebo. Empagliflozin increased plasma GDF-15 compared to placebo (adjusted between-groups treatment effect; ratio of change (1·09 [95% confidence interval (CI), 1.03–1.15]: p = 0.0040). The increase in plasma GDF15 was inversely associated with a decrease in left ventricular end-systolic (*R* = – 0.23, p = 0.031), and end-diastolic volume (*R* = – 0.29, p = 0.0066). There was no change in plasma hsCRP (1.09 [95%CI, 0.86–1.38]: p = 0.48) or plasma hsTNT (1.07 [95%CI, 0.97–1.19]: p = 0.18) compared to placebo. Patients with diabetes and treated with metformin demonstrated no increase in plasma GDF-15 with empagliflozin, p for interaction = 0·01.

**Conclusion:**

Empagliflozin increased plasma levels of GDF-15 in patients with HFrEF, with no concomitant increase in hsTNT nor hsCRP.

*Trial registration*: The Empire HF trial is registered with ClinicalTrials.gov, NCT03198585*.*

**Supplementary Information:**

The online version contains supplementary material available at 10.1186/s12933-022-01463-2.

## Background

Plasma growth differentiation factor 15 (GDF-15) is a member of the transforming growth factor β-superfamily, acting as a stress-responsive cytokine. The tissue expression of this biomarker is increased during several pathological conditions including inflammation, myocardial ischemia, and tissue injury [1, 2]. Increased plasma levels of GDF-15 are also associated with heart failure (HF), and linked to HF progression, adverse cardiac remodelling, long-term MACEs, serious cardiac arrhythmic events and mortality [3–6]. Elevated levels of GDF-15 are also associated with increased risk of sudden cardiac death within 24 h of an incident myocardial infarction [7] and are significantly correlated with an increased risk of lower extremity atherosclerotic disease in T2DM patients [8]. Other studies support the hypothesis that GDF-15 is more than a general stress induced cytokine, and increased level of plasma GDF-15 is considered as a novel therapeutic target in metabolic regulations (e.g. weight loss, improved insulin resistance, and systolic blood pressure reduction) [9–11].” Further, treatment with the glucose lowering drug metformin increased the circulating levels of GDF-15, and were associated with weight loss, improved glucose metabolism, and decreased appetite, which were correlated to the GDF-15 increase in patients with diabetes with or without obesity [12, 13].

Sodium glucose co-transporter (SGLT2) inhibitors improve clinical outcomes in both patients with diabetes, and/or with heart failure and reduced ejection fraction (HFrEF) [14–19]. Several studies have demonstrated beneficial cardiac, renal, and metabolic effects of SGLT2 inhibitors, where the cardiac and renal effects include cardiac reverse remodelling, reduction in filling pressures, blood pressure, and plasma volumes [20, 21]. Of the metabolic benefits, SGLT2 inhibitors demonstrated a reduction of blood glucose, body weight, and as well as an improved insulin resistance in HFrEF patients [22–24]. A recent proteomic study revealed an increase in plasma GDF-15 by empagliflozin in patients with diabetes or impaired glucose tolerance [25], however, it is unknown whether the SGL2Ti mediated improvement of HFrEF is accompanied by a change in plasma of GDF-15 levels.

In this current analysis of the Empagliflozin in Heart Failure Patients with Reduced Ejection Fraction (Empire HF) trial [26], we sought to evaluate the effect of empagliflozin on plasma GDF-15 along with the inflammatory marker high sensitive C-reactive protein (hsCRP), and marker of myocardial injury high sensitive troponin T (hsTNT) in patients with HFrEF.

## Methods

Empire HF trial [26] was designed, conducted and reported in accordance with a protocol in compliance with Good Clinical Practice and in accordance with the Declaration of Helsinki. All participants signed informed consent prior to inclusion. The Empire HF study protocol was approved by the relevant institutional review board (Danish National Committee on Health Research Ethics, number H-17010756).

### Study design

This present Empire HF Biomarker study was an exploratory study of the Empire HF trial, which has been described, and published [26, 27]. The trial was an investigator-initiated, multicentre, randomized, double-blind, placebo-controlled trial, in which HFrEF patients were randomly assigned to empagliflozin or matching placebo (1:1) for 12 weeks between June 29, 2017, through September 10, 2019.

### Study participants

A full list of inclusion and exclusion criteria is provided in appendix p3. Briefly, stable HFrEF patients aged ≥ 18 years, with New York Heart Association (NYHA) functional class I–III symptoms and left ventricular ejection fraction (LVEF) of 40% or less were eligible. Patients with known type 2 diabetes were treated in accordance with European and national guidelines and were required to have a glycated haemoglobin (HbA_1C_) level in the range of 48–83 mmol/mol (6.5–10.0%) and stable doses of anti-glycaemic treatment within the last 30 days. Exclusion criteria included symptomatic hypotension with a systolic blood pressure < 95 mmHg, estimated glomerular filtration rate ≤ 30 mL/min/1.73 m^2^, admission for HF within 30 days, or admission for hypoglycaemia in the past 12 months [26].

### Randomization

All study related procedures including blood samples were performed at two sites (Odense University Hospital, and Herlev and Gentofte University Hospital). Eligible patients were randomly assigned in a double-blind fashion using a fixed-randomization schedule, according to blocks of 10. Patients underwent an examination program at randomization, including clinical examination, blood tests, and were evaluated in the outpatient clinic after 45 ± 10 days for examination of volume status, and recording of adverse events. A blood test was performed to assess renal function and electrolyte levels. At follow-up, the investigation program was repeated after 12 weeks.

### Biomarkers

Blood tests were performed at baseline and repeated at 12 weeks follow-up. Plasma concentrations of GDF-15, and hsTNT were analysed on fasting blood samples at baseline and follow-up, which were immediately centrifuged upon collection and stored at − 80 °C. A batch analysis was performed at a central laboratory (GDF-15 Roche Elecsys Assay; Roche Diagnostics GmbH, Mannheim, Germany) [28]. Plasma concentrations of hsCRP were analysed on fasting blood samples at baseline and follow-up on the Atellica essay, and Cobas8000 platform with a coefficient variation < 10%.

### Echocardiography

Echocardiographic protocol has been previously described in detail [22]. In brief, transthoracic echocardiography was performed on a Vivid e9 ultrasound system (General Electric, Horten, Norway), and stored digitally for offline analysis. For two-dimensional and Doppler images, three consecutive beats for patients in sinus rhythm and multiple beats for patients with atrial fibrillation were measured and averaged. Left ventricular (LV) end systolic volume (LVESV), LV end-diastolic volume (LVEDV), and left atrial volume index (LAVi) were assessed using the biplane method of disks. LV dimensions were measured from frozen end-diastolic and end-systolic 2D images in the parasternal long axis to assess LV mass (LVM).

Plasma volume was estimated as: (1 − hematocrit) × (α + [β × weight in kg]), in which α = 1530 and β = 41 for men, whereas α = 864 and β = 47.9 for women [29, 30].

### Statistical analysis

Primary efficacy measure was the between-group difference in the change of plasma GDF-15 from baseline to 12 weeks follow-up. Secondary exploratory measures included the between-group differences in the changes of hsCRP and hsTNT.

No specific sample size estimation was performed for this post-hoc analysis, and study population size was the sample size of the main Empire HF trial [26]. The primary statistical analysis was based on all included patients with available plasma GDF-15 measurement based on the per protocol principle. We replicated the analyses with the full study cohort, and findings remained consistent with the per protocol analysis in a sensitivity analysis (Additional file 1: Online Appendix p4).

Plasma GDF-15 was log-transformed due to the skewed distribution. The between group difference in GDF-15 was analysed using a linear mixed effect model with a random intercept to account for repeated measurements from the same individual and reported as a ratio of change value with 95% confidence intervals (95% CIs) for the between-group changes.

For normally distributed measurements, the aforementioned statistical analysis was used but reported as a coefficient value with 95% CIs. To reduce the risk for false discoveries in a post-hoc study, we adjusted for age, sex, body mass index (BMI), estimated glomerular filtration (eGFR), and diabetes.

Pearson’s correlation coefficients, the corresponding two-sided 95%CIs, and p-values were calculated to examine the association between the change in the log-transformed plasma GDF-15 concentrations from baseline to 12 weeks and the following changes in the cardiac, renal, and metabolic measurements; LVESV, LVEDV, LAVI, LVM, systolic blood pressure, the metabolic parameters; weight, BMI, HbA_1C_, and the renal parameters; haematocrit, estimated glomerular filtration rate (eGFR), and plasma volume.

A two-sided significance level of 0.05 was considered significant. Data frequencies are expressed as mean standard deviation (SD) for normally distributed variables, number (%) for categorical variables, and median with interquartile range (IQR) for non-normally distributed variables. Statistical analyses were conducted using Stata Statistical Software, version 16 (Stata Corp, College. Station, Texas, USA). The Empire HF is publicly registered on ClinicalTrials.gov, NCT03198585, and EudraCT, 2017-001341-27.

## Results

**Fig. 1:**
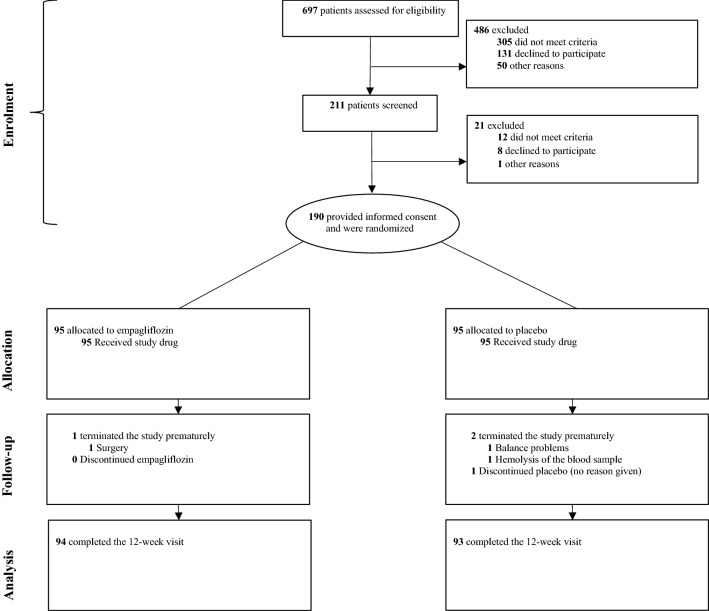
Trial profile

Between June 29, 2017, and September 10, 2019, 697 participants were assessed for study eligibility, of which 190 participants were randomly assigned to receive either empagliflozin (10 mg/day) (n = 95) or placebo (n = 95) for 12 weeks. For the plasma GDF-15 outcome, one (1%) patient in the intervention group wished no further follow-up due to surgery of condition unrelated to the study. In the placebo group, one (1%) patient terminated the study prematurely due to dizziness unrelated to the study. One (1%) patient had a blood sample which could not be analysed and were excluded from the analysis (Fig. 1). As a result, blood sample results for plasma GDF-15 were available in 94 patients in the empagliflozin group, and 93 patients in the placebo group at follow-up.

The groups were well-matched with respect to baseline characteristics, with a high proportion of patients receiving the guideline recommended pharmacologic therapy for HFrEF (Table 1). The mean age was 64 ± 11 years, 162 (85%) were male, 149 (78%) reported New York Heart Association class II functional status, 97 (51%) had ischemic HF as ethology, 36 (19%) had a cardiac resynchronization device, and 91 (48%) had an implantable cardioverter-defibrillator. Plasma N-terminal—pro Brain Natriuretic Peptide (NT-proBNP) was moderately elevated with a median of 591 (IQR 304–1048) pg/mL, 24 patients (13%) had type 2 diabetes with eight patients (4%) in treatment with metformin. The median adherence to the allocated treatment was 100 (IQR 99–100) %. One (1%) patient in the placebo group had a treatment adherence less than 80% (Fig. 1).

**Table 1:** Baseline characteristics

	Empagliflozin, 10 mg/day	Placebo
	(n = 94)	(n = 93)
Age (years)	65 (10)	63 (12)
Sex (male)	78 (83%)	81 (87%)
Caucasian (%)	91 (97%)	92 (99%)
Body mass index (kg/m^2^)	29 (26–32)	28 (26–33)
Smoking	22 (23%)	18 (19%)
Systolic blood pressure (mmHg)	119 (19)	121 (16)
Heart rate (beats/min)	69 (11)	72 (13)
Heart failure characteristics		
Duration of heart failure (months)	37 (12–69)	27 (13–62)
Heart failure cause		
Ischemic heart failure	48 (51%)	49 (53%)
Non-ischemic heart failure	46 (49%)	44 (47%)
Latest recorded ejection fraction (%)	29 (8)	30 (7)
NYHA class		
I	5 (5%)	7 (8%)
II	71 (76%)	77 (83%)
III	18 (19%)	9 (10%)
KCCQ Overall Summary Score	79 (65–90)	78 (65–90)
KCCQ Clinical Summary Score	82 (68–95)	81 (68–92)
Comorbidities		
Type 2 diabetes	11 (12%)	12 (13%)
Hypertension	35 (37%)	40 (43%)
Atrial fibrillation	33 (35%)	32 (34%)
Ischemic heart disease	50 (53%)	53 (57%)
Chronic kidney disease^a^	13 (14)	12 (13)
Laboratory variables		
GDF-15 (pg/mL)	1189 (918–1720)	1299 (952–1823)
In ischemic heart failure	1110 (742–1820)	1397 (1020–2090)
In non-ischemic heart failure	1230 (977–1607)	1205 (851–1650)
NT-proBNP (ng/L)	565 (303–1020)	605 (334–1060)
In sinus rhythm	408 (276–655)	493 (244–793)
In atrial fibrillation	1050 (596–1820)	960 (566–1470)
Estimated glomerular filtration rate (mL/min/1.73 m^2^)	73 (57–89)	74 (60–90)
Haemoglobin A1c (mmol/mol)	40 (36–43)	39 (36–42)
Haematocrit (%)	41 (38–44)	41 (39–45)
Heart failure medication		
ACE inhibitors/ARBs	58 (62%)	63 (68%)
Sacubitril–valsartan	30 (32%)	27 (29%)
β blockers	89 (95%)	87 (94%)
Mineralocorticoid-receptor antagonist	61 (65%)	61 (66%)
Diuretics^b^	62 (66%)	60 (65%)
Device type		
Cardiac resynchronization therapy without ICD	7 (14%)	4 (8%)
Cardiac resynchronization therapy with ICD	11 (22%)	12 (24%)
ICD only	33 (65%)	31 (62%)
Patients with a history of type 2 diabetes		
Metformin	4 (36%)	4 (33%)
Sulfonylurea	2 (18%)	0 (0%)
DPP-4 inhibitor	0 (0%)	5 (42%)
GLP-1 receptor agonist	0 (0%)	1 (8%)
Insulin	5 (45%)	3 (25%)

Values are presented as mean (SD), median interquartile range (IQR) or number (%). There were no significant differences between the groups

*NYHA* New York Heart Association, *KCCQ* Kansas City Cardiomyopathy Questionnaire, *GDF15* Plasma growth differentiation factor 15, *NT-proBNP* N-terminal pro–B-type natriuretic peptide, *ACE* inhibitors, angiotensin-converting enzyme inhibitors, *ARB* angiotensin-II-receptor blockers, *ICD* implantable cardioverter defibrillator

^a^Chronic kidney disease was defined with an addition to an estimated glomerular filtration rate under 60 mL/min/1.73 m^2^

^b^Diuretics includes Loop diuretics or Thiazide

The median plasma GDF-15 level was 1218 (IQR 918–1820) pg/mL (Table 1). Baseline plasma levels of GDF-15 were associated with the baseline plasma levels of hsCRP (*R* = 0.22, p = 0.0020), hsTNT (*R* = 0.32, p < 0.0001), and NT-proBNP (*R* = 0.37, p < 0.0001).

**Fig. 2:**
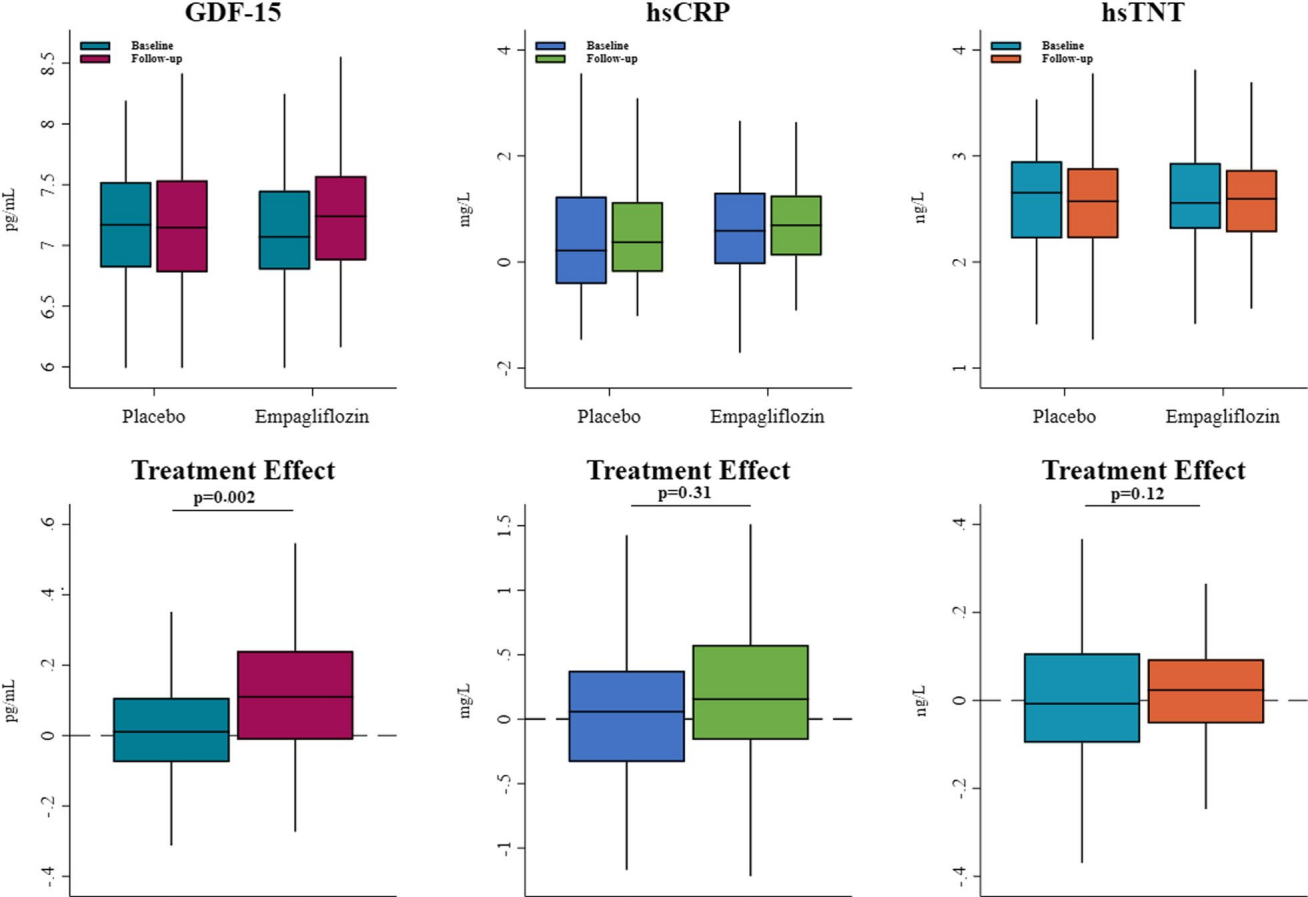
Values at baseline and follow-up by group for each variable and Treatment effects. Unadjusted values at baseline and 12 weeks follow-up, and the unadjusted treatment effect in the empagliflozin and placebo group with the adjusted p value. The box represents median with IQR, and whiskers represent 1.5 times the IQR. *GDF-15* growth differentiation factor 15, *HsCRP* high-sensitivity C-reactive protein, *HsTNT* high-sensitivity Troponin T

**Fig. 3:**
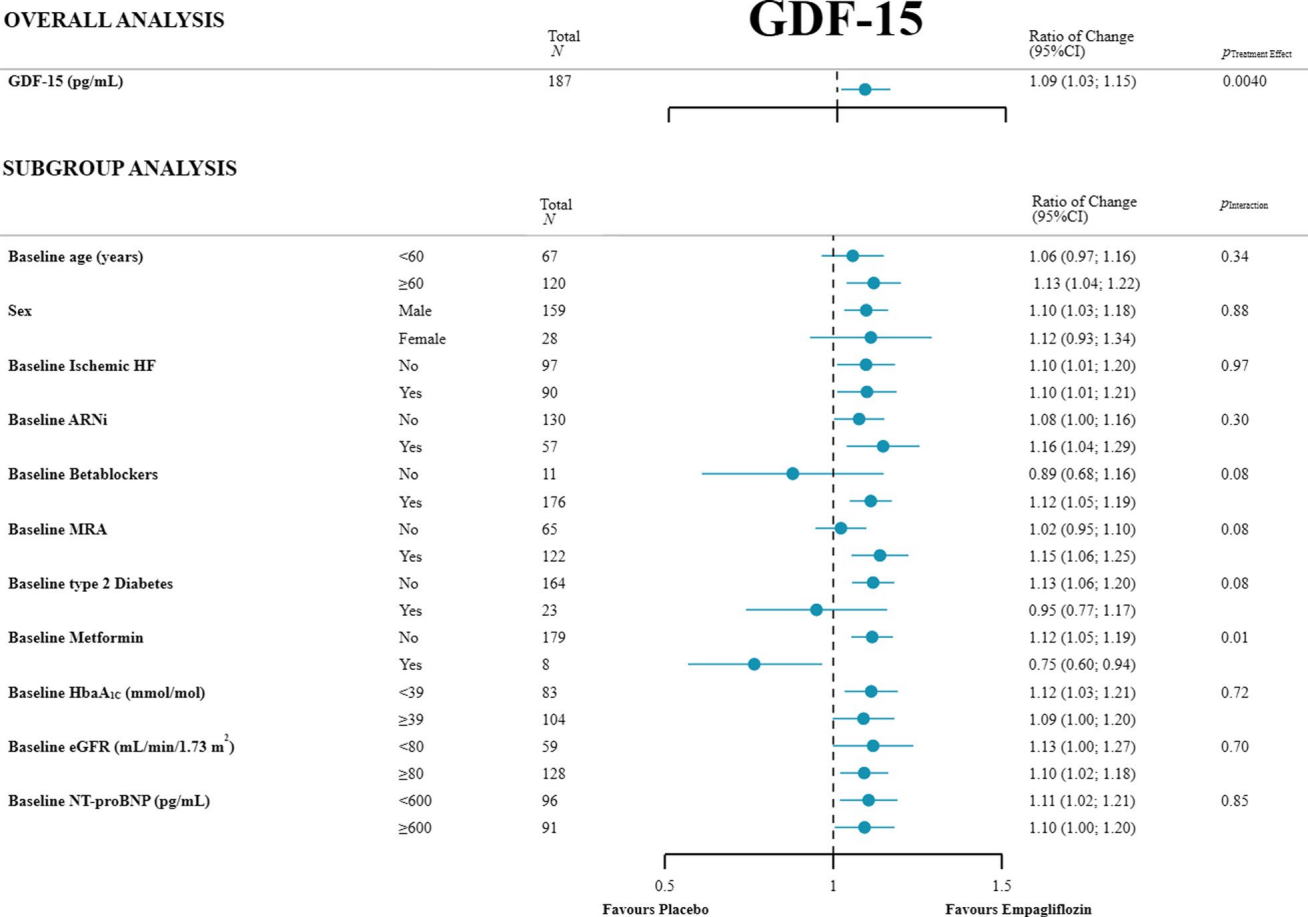
Subgroup analyses of GDF-15. Mean (95% CI) change in the empagliflozin group versus the placebo group. For continuous variables, the cut-off value is illustrated in the figure. *GDF-15* growth differentiation factor 15, *HF* heart failure, *ARNi* angiotensin receptor-neprilysin inhibitor, *MRA* mineralocorticoid receptor antagonist, *NT-proBNP* N-terminal pro-B-type natriuretic peptide

Patients treated with empagliflozin experienced a statistically significant (9%) increase in plasma GDF-15 compared to placebo (adjusted ratio of change: 1.09 [95% confidence interval (CI), 1.03 to 1.15]: p = 0.0040). Median plasma GDF-15 was 1189 (918–1720) pg/mL at baseline, and 1394 (970–1942) pg/mL at 12 weeks with Empagliflozin. Placebo: median plasma GDF-15 at baseline and at 12 weeks was baseline 1299 (952–1823) pg/mL, and 1271 (879–1872) pg/mL, respectively (Table 2; Fig. 2). The empagliflozin-mediated increase in plasma GDF-15 was consistent across multiple subgroups including sex, HF ethology, congestive HF medication, and NT-proBNP (Fig. 3). Median plasma GDF-15 was 1980 (1434–2608) pg/mL in the 23 (12%) patients with diabetes compared to those without 1169 (861–1632) pg/mL, with a borderline tendency towards no effect in patients with diabetes, p for interaction 0.08. In the small group of patients with diabetes and who were treated with metformin there was no increase in plasma GDF-15 with empagliflozin, p for interaction = 0.01 (Fig. 3). The increase in plasma GDF-15 from baseline to 12 weeks by empagliflozin was inversely correlated with a decrease in LVESV (*R* = – 0.23, p = 0.031), and LVEDV (*R* = – 0.29, p = 0.0066), with a significant between-group difference association to the change in GDF-15 (Table 3). There was no association between the increase in plasma GDF-15 and the decreases in LAVI, or LVM (Table 3). Finally, there was a borderline significant association between the increase in plasma GDF-15 with the decrease in systolic blood pressure from baseline to follow-up (*R* = – 0.20, p = 0.052) (Table 3).

**Table 2:** Changes in efficacy measures

	Empagliflozin, 10 mg/day	Placebo	p value
*Outcome measurements*			
**GDF-15 (pg/mL)**			
Baseline	1188 (918 to 1720)	1299 (952 to 1823)	
At 12 weeks	1394 (970 to 1942)	1271 (879 to 1872)	
Change over 12 weeks	124 (– 27 to 297)	12 (– 87 to 156)	
Adjusted between group treatment effect^a^	1.09 (1.03 to 1.15)		0.0040
**hsCRP (mg/L)**			
Baseline	1.82 (0.96 to 3.70)	1.24 (0.66 to 3.40)	
At 12 weeks	2.00 (1.13 to 3.50)	1.43 (0.83 to 3.05)	
Change over 12 weeks	0.20 (– 0.30 to 1.20)	0.05 (– 0.50 to 0.50)	
Adjusted between group treatment effect^a^	1.09 (0.86 to 1.38)		0.48
**hsTNT (ng/L)**			
Baseline	12.90 (10.10 to 18.30)	14.20 (9.22 to 19.00)	
At 12 weeks	13.40 (9.79 to 17.60)	13.10 (9.25 to 17.90)	
Change over 12 weeks	0.33 (– 0.90 to 1.00)	– 0.10 (– 1.26 to 1.03)	
Adjusted between group treatment effect^a^	1.07 (0.98 to 1.19)		0.18
*Related measurements*			
*Cardiac*			
**LVESV (mL)**			
Baseline	108.09 (52.04)	102.43 (45.05)	
At 12 weeks	99.98 (45.52)	101.90 (44.55)	
Change over 12 weeks	– 8.50 (26.45)	0.01 (31.32)	
Adjusted between group treatment effect	– 8.79 (– 17.39 to – 0.19)		0.045
*Metabolic*			
**BMI (kg/m**^**2**^**)**			
Baseline	28.94 (26.48 to 32.42)	28.27 (25.86 to 32.96)	
At 12 weeks	28.68 (26.28 to 31.97)	28.78 (26.04 to 32.64)	
Change over 12 weeks	– 0.33 (– 0.69 to – 0.03)	0.15 (− 0.35 to 0.48)	
Adjusted between group treatment effect^a^	– 0.37 (– 0.57 to – 0.18)		< 0.0001
*Renal*			
**Plasma volume (mL)**			
Baseline	3032.59 (404.59)	3175.05 (456.44)	
At 12 weeks	2897.51 (383.44)	3177.65 (415.58)	
Change over 12 weeks	– 135.08 (135.69)	7.7 (144.25)	
Adjusted between group treatment effect	– 115.04 (– 152.15 to – 77.93)		< 0.0001

Median values with interquartile range are represented for variables with skewed data

*GDF-15* growth/differentiation factor 15, *hsCRP* high sensitive C-reactive protein, *HsTNT* high-sensitivity troponin T, *LVESV* left ventricular end-systolic volume, *BMI* body mass index

^a^Present the between-group treatment effect as a ratio of change. Adjusted for age, sex, and type 2 diabetes

**Table 3:** Correlations of the change in GDF-15 with the change in other variables at 12 weeks

	Empagliflozin, 10 mg/d		Placebo		Between-group difference
	*R*	*p* value	*R*	*p* value	*p* value
*Cardiac measurements*					
LVESV (mL)	− 0.23	0.031	0.099	0.37	0.035
LVEDV (mL)	− 0.29	0.0066	0.12	0.29	0.011
LVM (g/m^2^)	− 0.10	0.32	0.03	0.77	0.36
LAVI (ml/m^2^)	− 0.02	0.86	0.05	0.64	0.64
SBP (mmHg)	− 0.20	0.052	− 0.12	0.24	0.50
*Metabolic measurements*					
Weight (kg)	− 0.08	0.418	− 0.24	0.020	0.13
HbA1c (mmol/L)	0.08	0.429	− 0.15	0.147	0.096
BMI (kg/m^2^)	− 0.10	0.394	− 0.26	0.012	0.12
*Renal measurements*					
Haematocrit (%)	− 0.04	0.679	− 0.03	0.76	0.94
Plasma volume (mL)	0.01	0.887	− 0.07	0.498	0.55
eGFR (mL/min/1·73 m^2^)	− 0.42	< 0.0001	− 0.53	< 0.0001	0.68

Pearson’s correlation between the change in GDF-15 and other variables. The increase in plasma GDF15 was inversely associated with the decrease in LVESV and LVEDV

*LVESV* left ventricular end-systolic volume, *LVEDV* left ventricular end-diastolic volume, *LVM* left ventricular mas, *LAVi* left atrial volume index, *SBP* systolic blood pressure, *HbA*_*1c*_ haemoglobin A_1c_, *BMI* body mass index, *eGFR* estimated glomerular filtration rate

The increase in plasma GDF-15 from baseline in empagliflozin recipients was unrelated to weight loss (*R* = – 0.10, p = 0.42), or BMI (*R* = – 0.089, p = 0.39) (Tables 2, 3). There was no significant correlation between the increase in plasma GDF-15 and the decrease in plasma volume (*R* = 0.015, p = 0.89), the decrease HbA1c (*R* = 0.14, p = 0.18), or the increase in haematocrit (*R* = – 0.043, p = 0.68) (Tables 2, 3).

There were no significant changes in the inflammatory biomarker plasma hsCRP (adjusted ratio of change: 1.09 [95%CI, 0.86 to 1.38]: p = 0.48), nor in hsTNT (adjusted ratio of change: 1.09 [95%CI, 0.97 to 1.19]: p = 0.18) in patients treated with empagliflozin compared to placebo (Table 2). No significant interaction was observed across different baseline characteristics in the subgroup analyses for plasma hsCRP or hsTNT (Additional file 1: Online Appendix).

We found no significant interaction.

## Discussion

Empagliflozin significantly improves cardiovascular outcomes in HFrEF patients regardless of diabetic status [31], with beneficial effects on the cardiac, renal, and metabolic functions [22, 24]. In this post-hoc study of 187 HFrEF patients predominantly without diabetes, we found that treatment with empagliflozin led to an 9% increase in plasma GDF-15 compared to placebo after 12 weeks of treatment. The increase of GDF-15 was inversely correlated with the decrease in LVESV, and LVEDV. Importantly, no concomitant changes were found in the inflammatory biomarker hsCRP or plasma hsTNT, a marker of myocardial injury.

GDF-15 is considered as an inflammatory biomarker of cell stress linked to cardiovascular disease in response to myocardial tissue injury [32, 33]. Increased levels of plasma GDF-15 have been associated with increased pulmonary capillary wedge pressure (cardiac filling pressure), increased LVM, and decreased LV systolic function after adjustment for traditional cardiovascular risk factors [3, 21]. Therefore, it is tempting to speculate that the elevated plasma GDF-15 in the present study is a consequence of deterioration in LV function, inflammation, and/or myocardial injury. This study demonstrated a significant inverse correlation between the increase in plasma GDF-15 and the decrease in LVESV and LVEDV from baseline to follow-up. Moreover, circulating hsTNT was not increased by treatment with empagliflozin for 12 weeks, and there was no association between the increase in plasma GDF-15 and the change in plasma hsTNT from baseline.

Although increased GDF-15 levels have shown to be associated with HF severity in patients with HFrEF, as far as we know, no HF medication have been shown to change the levels of GDF-15. An analysis of the PARADIGM-HF trial showed that GDF-15 levels were not modified by sacubitril/valsartan and was strongly associated with mortality and cardiovascular outcome [34]. Therefore, the pathological increase in GDF-15 should be distinguished from medication induced changes in GDF-15 as it has been described for Metformin, and now suggested for SGLT2 inhibitors in this study, by empagliflozin. Whether the empagliflozin induced GDF-15 increase is associated with the beneficial outcomes is speculative and needs further clarification in future studies. However, given the findings in this study, it is unlikely that the increase in plasma GDF-15 by empagliflozin was triggered by myocardial injury or deterioration in cardiac function. “Other studies have demonstrated increased levels of plasma GDF-15 to be more than an inflammatory cytokine, and suggested elevated GDF-15 expression and circulating levels act as a beneficial metabolic regulator, which correlate with weight loss, improved glucose tolerance, reduced food intake and appetite [35, 36]. A recent study demonstrated significantly increased levels of plasma GDF-15 by metformin treatment [35, 37], where they reported that weight loss, improved glucose metabolism, and decreased appetite were correlated to the GDF-15 increase. Importantly, the study demonstrated that metformin’s ability to lower weight, circulating glucose, and fasting insulin levels was restrained in GDF-15 knock-out mice, suggesting a GDF-15 mediated mechanism by metformin.

Same metabolic improvements have been reported in patients treated with SGLT2 inhibitors not only in diabetes, but also in HFrEF patients [21, 22]. The link between SGLT2 inhibitors and weight loss is not fully understood, but theories include urinary glucose loss with concomitant sodium excretion, subsequent improvement in glucose metabolism, and weight loss without a compensatory food intake [21]. We did not observe a significant association between the empagliflozin induced metabolic benefits; weight loss, and the increase in plasma GDF-15. Although this study is a post-hoc analysis, it was tested on 190 patients, and a strong association would have been detected. But several possible reasons might explain the lack of the association. First, compared to our 9% increase in plasma GDF-15 after 12 weeks of treatment, metformin increased plasma GDF-15 by 48% after 24 weeks of treatment. Metformin might therefore be a more potent drug on the GDF-15 regulation than empagliflozin [35], or the result could be explained by a longer treatment period with metformin. Second, our interaction analysis revealed tendency toward no increase in plasma GDF-15 in patients with diabetes, and significant for those few patients who were on metformin treatment. This finding provides a hypothesis that empagliflozin might act with a similar mechanism of action as metformin, and thus the modest increase in GDF-15 may explain the lack of significance in the metabolic regulators. However, the prevalence of diabetes was too low, and the statistical interaction analyses performed should be interpreted with caution. Third, we showed that empagliflozin demonstrated a significant reduction in lean mass, but not in fat mass, fat percentage, or visceral adipose tissue in HFrEF patients [21], which may further explain the lack of the correlation with weight loss in this study. The ability of GDF-15 to produce metabolic benefits by improving insulin resistance [38] may explain the improvement in insulin resistance that was induced by empagliflozin in patients with HFrEF without concomitant diabetes [21]. Finally, the patients in the present study were mainly without diabetes, and the effects of empagliflozin on plasma GDF-15 may be different in populations of HFrEF patients with a higher prevalence of diabetes and/or in severe HFrEF patients.

Although this study is a post-hoc analysis, the strength and consistency of the observed effects of empagliflozin treatment on plasma GDF-15 across participants with different baseline characteristics adds to the generalizability of the results. There are further limitations to our study design that justify acknowledgment here. Present population was predominantly without diabetes, and extrapolation of the findings to patients with diabetes and HFrEF should be done with caution. This study is a short-term trial, and whether the increase in plasma GDF-15 is sustained, or even further increased with longer treatment is speculative. More generally, there are increased risk for false discoveries in a post-hoc study; to account for this risk in our methods, we applied multiplicity adjustment.

## Conclusion

In conclusion, empagliflozin significantly increased the levels of plasma GDF-15 in a mainly non-diabetic HFrEF population after 12 weeks of treatment. The increase in plasma GDF-15 was unrelated to circulating levels of hsCRP, or hsTNT. The observed effect of empagliflozin on plasma GDF-15, and especially in an HFrEF population adds novelty to the existing literature. Our finding should raise the intriguing reconsideration of GDF-15’s mechanisms of action by empagliflozin in this population. The mechanisms underlying the empagliflozin-mediated increase in plasma GDF-15 remains to be established, and whether long-term treatment with empagliflozin results in significant changes on the metabolic regulators remains to be investigated.

## Supplementary Information

**Additional file 1: Online Appendix:** **Table S1**: Full list of inclusion and exclusion criteria. **Table S2**: Treatment effect on the intention-to-treat population. The treatment effect was analysed using a linear mixed effect model with a random intercept to account for repeated measurements from the same individual, and reported as a ratio of change value (due to the skewed distribution) with 95% confidence intervals (95% CIs) for the between-group changes with age, sex, body mass index (BMI), estimated glomerular filtration (eGFR), and diabetes at baseline as covariates. The adjusted between-group treatment ratio of change effect on GDF-15, hsCRP, and hsTNT are listed below. **Fig. S1**. Subgroup analysis of hsCRP. Mean (95% CI) change in the empagliflozin group versus the placebo group. For continuous variables, the cut-off value is illustrated in the figure. HsCRP, high-sensitivity C-Reactive Protein; HF, heart failure; ARNi, angiotensin receptor-neprilysin inhibitor; MRA, mineralocorticoid receptor antagonist; NT-proBNP, N-terminal pro-B-type natriuretic peptide. **Fig. S2**. Subgroup analysis of hsTNT. Mean (95% CI) change in the empagliflozin group versus the placebo group. For continuous variables, the cut-off value is illustrated in the figure. HsTNT, high-sensitivity Troponin T; HF, heart failure; ARNi, angiotensin receptor-neprilysin inhibitor; MRA, mineralocorticoid receptor antagonist; NT-proBNP, N-terminal pro-B-type natriuretic peptide.

## Data Availability

The data that support the findings of this study are available on request from the corresponding author. The data are not publicly available because they contain information that could compromise the privacy of research participants.

## Abbreviations

GDF-15: Growth differentiation factor-15
hsCRP: High-sensitive C-reactive protein
hsTNT: High-sensitive troponin T
SGLT2: Sodium-glucose cotransporter-2
HFrEF: Heart failure with reduced ejection fraction
