# Supplementary material for: The effect of empagliflozin on growth differentiation factor 15 in patients with heart failure: a randomized controlled trial (Empire HF Biomarker)
Source: Cardiovasc Diabetol. 2022 Feb 27;21:34. doi: 10.1186/s12933-022-01463-2 (PMC8882292; doi:10.1186/s12933-022-01463-2)
Supplement: Supplementary file 1 — Additional file 1: Online Appendix: Table S1: Full list of inclusion and exclusion criteria. Table S2: Treatment effect on the intention-to-treat population. The treatment effect was analysed using a linear mixed effect model with a random intercept to account for repeated measurements from the same individual, and reported as a ratio of change value (due to the skewed distribution) with 95% confidence intervals (95% CIs) for the between-group changes with age, sex, body mass index (BMI), estimated glomerular filtration (eGFR), and diabetes at baseline as covariates. The adjusted between-group treatment ratio of change effect on GDF-15, hsCRP, and hsTNT are listed below. Fig. S1. Subgroup analysis of hsCRP. Mean (95% CI) change in the empagliflozin group versus the placebo group. For continuous variables, the cut-off value is illustrated in the figure. HsCRP, high-sensitivity C-Reactive Protein; HF, heart failure; ARNi, angiotensin receptor-neprilysin inhibitor; MRA, mineralocorticoid receptor antagonist; NT-proBNP, N-terminal pro-B-type natriuretic peptide. Fig. S2. Subgroup analysis of hsTNT. Mean (95% CI) change in the empagliflozin group versus the placebo group. For continuous variables, the cut-off value is illustrated in the figure. HsTNT, high-sensitivity Troponin T; HF, heart failure; ARNi, angiotensin receptor-neprilysin inhibitor; MRA, mineralocorticoid receptor antagonist; NT-proBNP, N-terminal pro-B-type natriuretic peptide. [file 12933_2022_1463_MOESM1_ESM.docx]

Cardiovascular diabetology

**Supplementary appendix**

This appendix has been provided by the authors to give readers additional information about their work.

Supplement to: Omar M, Jensen J, Kistorp C, et al. *The effect of Empagliflozin on Growth Differentiation Factor 15 in Patients with Heart Failure: a randomized controlled trial. Empire HF Biomarker*

**Supplementary appendix, Section A-D**

**Supplement to:**

Omar M, Jensen J, Kistorp C, et al. The effects of Empagliflozin on Growth Differentiation Factor 15 in Patients with Heart Failure and Reduced Ejection Fraction: a post-hoc analysis from the Empire HF trial

**Corresponding author**: Massar Omer, MD, PhD & Professor Jacob Eifer Møller, PhD, DSc

**TABLE OF CONTENT**

Section A: List of investigators and steering committee1

Section B: Manufactured product Section2

Section C: Full list of inclusion and exclusion criteria3

Section D: Treatment effect on per protocol population4

Section E: Subgroup analysis of hsCRP and hsTNT5

**Section A: List of investigators and steering committee**

**Investigators at Odense University Hospital, Odense, Denmark**

Principal investigator: Professor Jacob Eifer Møller, PhD, DSc

Sub-investigator: Massar Omar, MD

**Investigators at Herlev and Gentofte University Hospital, Herlev, Denmark**

Principal investigator & sponsor: Professor Morten Schou, PhD

Sub-investigator: Jesper Jensen, PhD

**Steering committee:**

Lars Køber MD (chair), Jacob Eifer Møller MD, Morten Schou MD, Massar Omar MD, Jesper Jensen MD, Caroline Kistorp MD, Mikael Kjær Poulsen MD, Christian Tuxen MD, Ida Gustafsson MD, Finn Gustafsson MD, Emil Fosbøl MD, Niels Eske Bruun MD, Lars Videbæk MD.

**Section B: Manufactured product & Safety Variables**

**Primary Object**

Adult male and female patients with chronic heart failure and reduced ejection fraction.

**Manufactured product, doses and mode of administration**

To the existing standard of care treatment for heart failure, empagliflozin 10 mg or a matching placebo was administered orally once daily for 12 weeks period. The manufacture of empagliflozin was not involved nor funded the study.

**Section C: Full list of inclusion and exclusion criteria**

| **Inclusion Criteria** | **Exclusion Criteria** |
| --- | --- |
| Optimal HF therapy in accordance with European and national guidelines | CRT-D/-P implanted < 90 days |
| LVEF ≤ 0.40 | Uncorrected severe valvular heart disease |
| Estimated GFR > 30 ml/min/1.73 m^2^ | Non-compliance |
| BMI < 45 kg/m^2^ | Use of metalozone |
| NYHA functional class I-III | NYHA functional class IV |
| Age > 18 years | Age > 85 years |
|  | Dementia |
|  | Hospitalisation for HF < 30 days |
| If T2D – optimal treatment in accordance with European and national guidelines | Hospitalisation for hypoglycaemia < 12 months |
| If T2D – stable doses of antidiabetic treatment for 30 days | Known sustained ventricular tachycardia |
| If T2D – HbA1c 6.5–10.0% (48–83 mmol/mol) | Symptomatic hypotension and systolic blood pressure < 95 mmHg |
|  | Unable to perform an exercise test |
|  | Immobilization |
|  | Pregnancy |
|  | Participation in other medical trials |
|  | Previous intolerance of empagliflozin or excipients |

**Section D: Treatment effect on the intention-to-treat population**

This sensitivity analysis included every subject who was randomized according to randomized treatment assignment in an intention-to-treat analysis on all the 190 HFrEF patients.

|  | **Empagliflozin, 10 mg/d** | |  | **Placebo** | ***P* value** |
| --- | --- | --- | --- | --- | --- |
| ***Outcome measurements*** | | |  |  |  |
| **GDF-15 (pg/mL)** |  | |  |  |  |
| Baseline | 1177 (899 to 1720) | |  | 1299 (915 to 1849) |  |
| At 12 weeks | 1394 (970 to 1942) | |  | 1271 (879 to 1872) |  |
| Change over 12 weeks | 124 (–27 to 297) | |  | 12 (–87 to 156) |  |
| Adjusted between group treatment effect^*^ |  | 1.10 (1.04 to 1.17) | | | 0.0020 |
| **hsCRP (mg/L)** |  | |  |  |  |
| Baseline | 1.8 (0.96 to 3.7) | |  | 1.2 (0.7 to 3.4) |  |
| At 12 weeks | 2 (1.1 to 3.5) | |  | 1.5 (0.8 to 3.1) |  |
| Change over 12 weeks | 0.2 (–0.3 to 1.2) | |  | 0.04 (–0.4 to 0.5) |  |
| Adjusted between group treatment effect^*^ |  | 1.13 (0.89 to 1.43) | | | 0.31 |
| **hsTNT (ng/L)** |  | |  |  |  |
| Baseline | 12.9 (10.1 to 18·8) | |  | 14.2 (9.2 to 19.1) |  |
| At 12 weeks | 13.4 (9.8 to 17·6) | |  | 13.1 (9.3 to 17.9) |  |
| Change over 12 weeks | 0.3 (–0.9 to 1) | |  | –0.1 (–1.3 to 1.0) |  |
| Adjusted between group treatment effect^*^ |  | 1.09 (0.98 to 1.20) | | | 0.12 |

The treatment effect was analysed using a linear mixed effect model with a random intercept to account for repeated measurements from the same individual, and reported as a ratio of change value (due to the skewed distribution) with 95% confidence intervals (95% CIs) for the between-group changes with age, sex, body mass index (BMI), estimated glomerular filtration (eGFR), and diabetes at baseline as covariates.

The adjusted between-group treatment ratio of change effect on GDF-15, hsCRP, and hsTNT are listed below.

**Section E: Subgroup analyses of hsCRP and hsTNT**

**
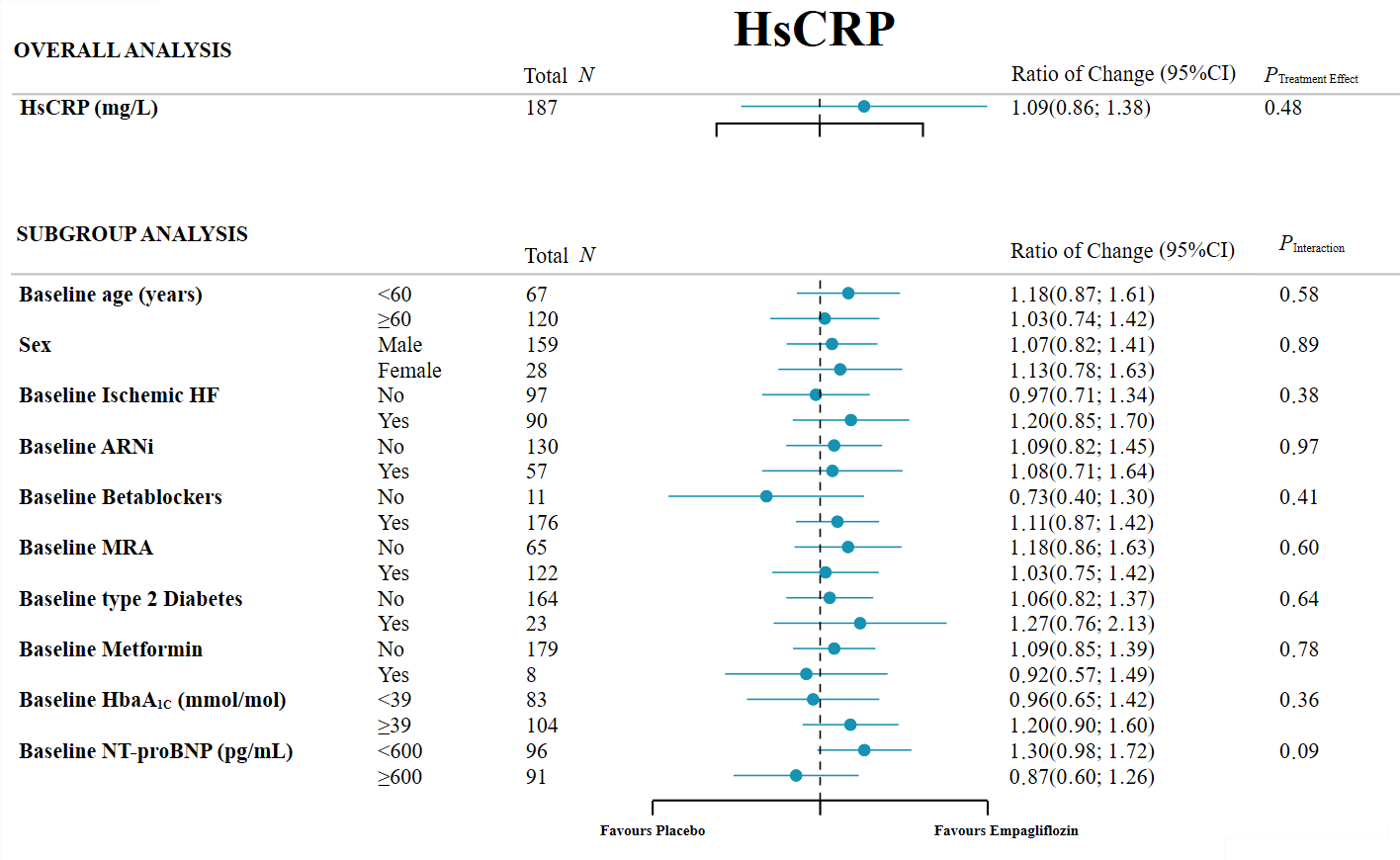
Subgroup analyses of hsCRP**

Mean (95% CI) change in the empagliflozin group versus the placebo group. For continuous variables, the cutoff value is illustrated in the figure.

HsCRP, high-sensitivity C-Reactive Protein; HF, heart failure; ARNi, angiotensin receptor-neprilysin inhibitor; MRA, mineralocorticoid receptor antagonist; NT-proBNP, N-terminal pro-B-type natriuretic peptide.

**
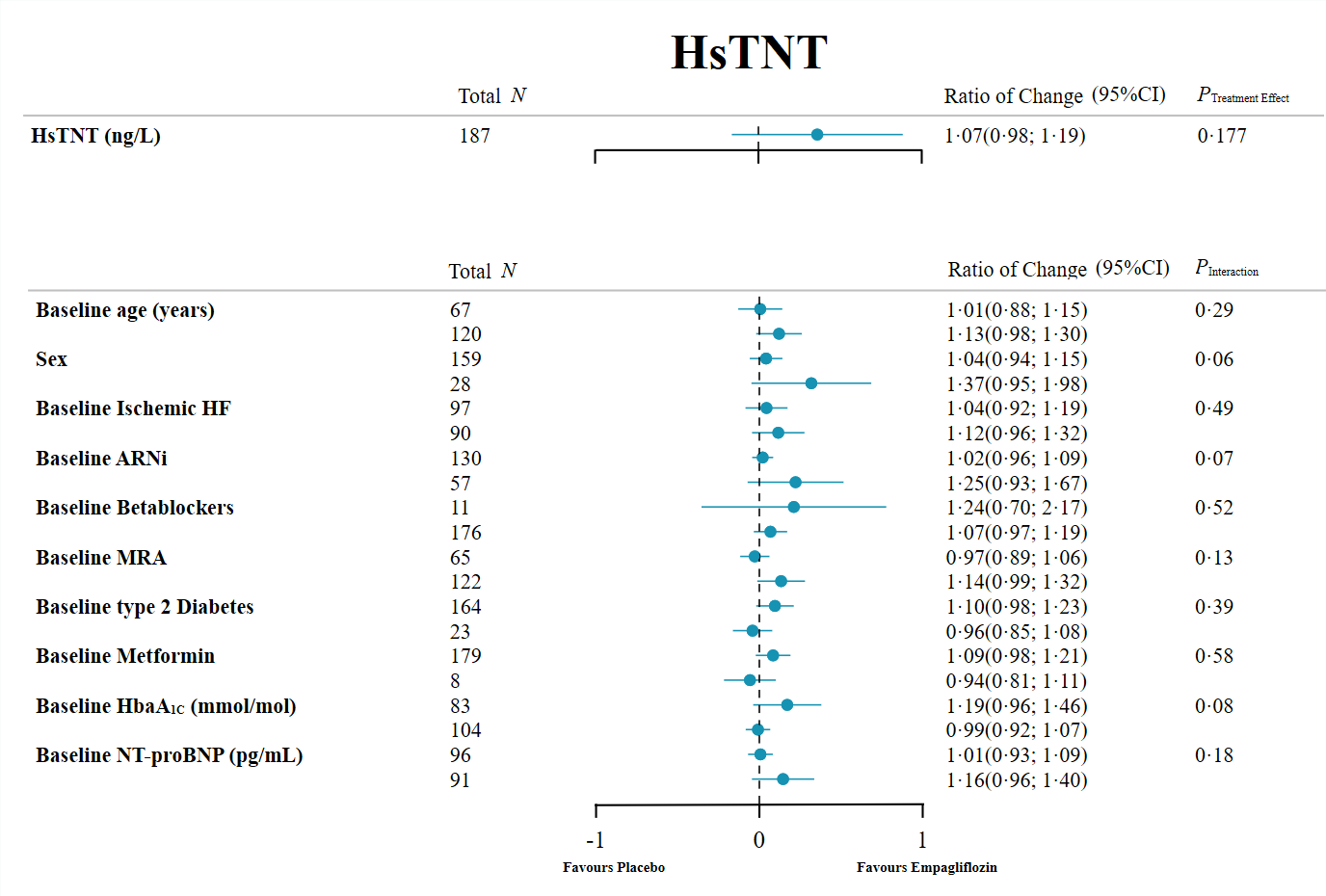
Subgroup analyses of hsTNT**

Mean (95% CI) change in the empagliflozin group versus the placebo group. For continuous variables, the cutoff value is illustrated in the figure.

HsTNT, high-sensitivity Troponin T; HF, heart failure; ARNi, angiotensin receptor-neprilysin inhibitor; MRA, mineralocorticoid receptor antagonist; NT-proBNP, N-terminal pro-B-type natriuretic peptide.

**Section F: Changes in efficacy measures on cardiac, metabolic and renal measurements**

| **Changes in efficacy measures** | | | | | |  |
| --- | --- | --- | --- | --- | --- | --- |
|  | **Empagliflozin, 10 mg/d** | | **Placebo** | | **p value** | |
| ***Related measurements*** | |  | |  | |  |
| ***Cardiac*** | |  | |  | |  |
| **LVEDV (mL)** |  | |  | |  | |
| Baseline | 162·14 (63·48) | | 157·81 (58·19) | |  | |
| At 12 weeks | 153·87 (56·83) | | 159·85 (57·19) | |  | |
| Change over 12 weeks | –8·86 (30·41) | | 2·29 (39·50) | |  | |
| Adjusted between group treatment effect | –10·91 (–21·40 to –0·42) | | | | 0.041 | |
| **LVM (g/m^2^)** |  | |  | |  | |
| Baseline | 261.03 (101.90) | | 268.09 (101.33) | |  | |
| At 12 weeks | 251.66 (111.08) | | 275.02 (91.10) | |  | |
| Change over 12 weeks | –9.37 (58.45) | | 10.08 (60.10) | |  | |
| Adjusted between group treatment effect | –17.61 (–34.66 to –0.57) | | | | 0.043 | |
| **LAVi (ml/m^2^)** |  | |  | |  | |
| Baseline | 40.83 (19.08) | | 36.57 (12.98) | |  | |
| At 12 weeks | 40.07 (17.19) | | 37.24 (13.24) | |  | |
| Change over 12 weeks | –1.08 (7.90) | | 1.28 (8.24) | |  | |
| Adjusted between group treatment effect | –2.20 (–4.58 to 0.18) | | | | 0.070 | |
| **SBP (mmHg)** |  | |  | |  | |
| Baseline | 119 (18) | | 121 (16) | |  | |
| At 12 weeks | 115 (14) | | 121 (14) | |  | |
| Change over 12 weeks | –4.40 (14.84) | | 0.38 (12.78) | |  | |
| Adjusted between group treatment effect | –4.21 (–8.13 to –0.30) | | | | 0.035 | |
| ***Metabolic*** |  | |  | |  | |
| **Weight (kg)** |  | |  | |  | |
| Baseline | 90.56 (16.56) | | 93.67 (17.82) | |  | |
| At 12 weeks | 89.38 (16.14) | | 93.57 (16.70) | |  | |
| Change over 12 weeks | –1.18 (1.80) | | 0.12 (2.63) | |  | |
| Adjusted between group treatment effect^*^ | –1.16 (–1.78 to –0.53) | | | | <0.0001 | |
| **HbA1c (mmol/L)** |  | |  | |  | |
| Baseline | 40 (36 to 43) | | 39 (36 to 42) | |  | |
| At 12 weeks | 38.5 (36 to 41) | | 39 (36 to 42) | |  | |
| Change over 12 weeks | –1 (–3 to 0) | | 0 (−2 to 2) | |  | |
| Adjusted between group treatment effect^*^ | 0.97 (0.95 to 0.99) | | | | 0.015 | |
| ***Renal*** |  | |  | |  | |
| **eGFR (mL/min/m^2^)** |  | |  | |  | |
| Baseline | 73 (57 to 89) | | 74 (60 to 90) | |  | |
| At 12 weeks | 72 (59 to 88) | | 77 (62 to 89) | |  | |
| Change over 12 weeks | 0 (−5 to 4) | | 0 (−2 to 3) | |  | |
| Adjusted between group treatment effect^*^ | 0.99 (0.96 to 1.02) | | | | 0.55 | |
| **Haematocrit (%)** |  | |  | |  | |
| Baseline | 41.78 (4.26) | | 40.68 (4.29) | |  | |
| At 12 weeks | 43.83 (4.35) | | 40.60 (4.26) | |  | |
| Change over 12 weeks | −0.08 (2.34) | | 2.05 (2.40) | |  | |
| Adjusted between group treatment effect | 2.23 (1.54 to 2.91) | | | | <0.0001 | |

Median values with interquartile range are represented for variables with skewed data.

*Present the between-group treatment effect as a ratio of change. Adjusted for age, sex, and type 2 diabetes.

LVEDV, left ventricular end-diastolic volume; LVM, left ventricular mass; LAVi, left atrial volume index; SBP, systolic blood pressure; HbA_1c_, Hemoglobin A_1c_; eGFR, estimated glomerular filtration rate.
